# Never Cared for What They Do: High Structural Stability of Guanine-Quadruplexes in the Presence of Strand-Break Damage

**DOI:** 10.3390/molecules27103256

**Published:** 2022-05-19

**Authors:** Tom Miclot, Cécilia Hognon, Emmanuelle Bignon, Alessio Terenzi, Stéphanie Grandemange, Giampaolo Barone, Antonio Monari

**Affiliations:** 1 Department of Biological, Chemical and Pharmaceutical Sciences, University of Palermo, viale delle Scienze, Ed. 17, 90128 Palermo, Italy; tom.miclot@unipa.it (T.M.); alessio.terenzi@unipa.it (A.T.); 2 Université de Lorraine and CNRS, LPCT UMR 7019, F-54000 Nancy, France; cecilia.hognon@univ-lorraine.fr (C.H.); emmanuelle.bignon@univ-lorraine.fr (E.B.); 3 Université de Lorraine and CNRS, CRAN UMR 7039, F-54000 Nancy, France; stephanie.grandemange@univ-lorraine.fr; 4 Université Paris Cité and CNRS, ITODYS, F-75006 Paris, France

**Keywords:** guanine quadruplexes, DNA strand breaks, molecular modeling and simulation

## Abstract

DNA integrity is an important factor that assures genome stability and, more generally, the viability of cells and organisms. In the presence of DNA damage, the normal cell cycle is perturbed when cells activate their repair processes. Although efficient, the repair system is not always able to ensure complete restoration of gene integrity. In these cases, mutations not only may occur, but the accumulation of lesions can either lead to carcinogenesis or reach a threshold that induces apoptosis and programmed cell death. Among the different types of DNA lesions, strand breaks produced by ionizing radiation are the most toxic due to the inherent difficultly of repair, which may lead to genomic instability. In this article we show, by using classical molecular simulation techniques, that compared to canonical double-helical B-DNA, guanine-quadruplex (G4) arrangements show remarkable structural stability, even in the presence of two strand breaks. Since G4-DNA is recognized for its regulatory roles in cell senescence and gene expression, including oncogenes, this stability may be related to an evolutionary cellular response aimed at minimizing the effects of ionizing radiation.

## 1. Introduction

Guanine-quadruplex (G4) DNA or RNA structures can be produced by both intra-strand (i.e., produced by the folding of a single-stranded DNA fragment) or inter-strand G-G pairing and may adopt various topologies, depending on the orientation of the glycosidic bond, giving rise to parallel, antiparallel, and hybrid arrangements [1]. The rigid tetrad cores are connected by nucleotide loops, whose length and flexibility may exhibit rather large variations. The structural and dynamic properties of G4s have important and versatile biological implications. For example, DNA or RNA G4s may regulate viral infection cycles, becoming, as a consequence, potential therapeutic targets for antiviral drug candidates [2,3]. For this reason, they are also of major interest in the context of the current pandemic caused by the infectious pathogen SARS-CoV-2, whose genome has been shown to contain G4-compatible regions [4,5,6,7,8]. Interesting and recent reviews on the antiviral possibilities offered by G4 can be found in literature [9,10,11]. G4s are also involved in some neurological diseases, such as alpha-thalassemia or X-linked intellectual disability syndrome, in which they are positively or negatively involved in a cascade of gene expression regulation [12,13,14]. G4s are also involved in DNA replication pathways [15,16] and in gene expression, since they have been localized in oncogene and viral DNA promoter regions [17,18,19,20]. In addition, G4 are abundant in the terminal sequences of chromosomes, the telomeres, playing an important role in regulating the cellular life cycle by controlling replication-induced shortening of the telomeres, and hence, cellular programmed death via inhibition of telomerase [21,22,23,24]. As a matter of fact, the disruption of this mechanisms is linked to the immortality phenotype of cancer cells, which makes G4s ideal targets for cancer chemotherapeutic agents. Remarkably, G4s are also involved in conferring *Deinococcus radiodurans* its extraordinary resistance to ionizing radiation [25,26].

Because of their versatile and rather ubiquitous biological roles, several studies have focused on the effect of different DNA damage on the stability of G4s. In particular, oxidative damage has been scrutinized due to the fact that G4s are inherently composed of guanine-rich sequences, and the latter is the most easily oxidized nucleotide, and 8-oxo-guanine (8-OxoG) is its most common oxidation product [27,28,29,30]. Although G4s are clearly considered hotspots for oxidative DNA damage, they have shown strong structural resistance to this class of lesion [31], depending on the amount of oxidative lesions and their position in the DNA backbone [31,32,33,34].

**Figure 1:**
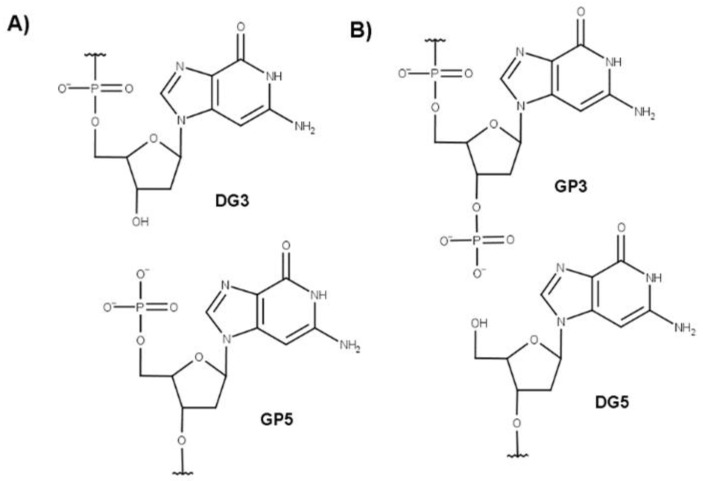
(**A**) Canonical and (**B**) non-canonical strand break damages occurring in the phosphodiester -O-P(O2)-O- bond of a nucleic acid.

Besides guanine modification or deletion, oxidative stress [35,36] and ionizing radiation [37] are also able to induce DNA strand-break damage. Strand breaks may occur at two different points of the same DNA strand, leading to a very strong genome instability, usually resulting in cell death. The resulting highly toxic damage is essentially due to the difficulty in repairing the dispersed DNA fragments. Interestingly, radiation-resistant bacteria possess specific DNA binding proteins that colocalize at the lesion foci favoring, in this way, their repair [38]. Ionizing radiation can result in two kinds of strand-breaks: canonical 5′-PO_4_^−^/3′-OH (CA), and non-canonical 5′-OH/3′-PO_4_^−^ (NC) [39,40], as shown in Figure 1. Both kinds of DNA terminations are known to occur in biological systems, produced by the activity of deoxyribonucleases I and II [41], respectively. However, the two types of damages are not equivalent: CA strand breaks are also commonly produced by normal cellular processes, notably during replication and repair [42], and thus are more easily recognized by DNA ligase, which may catalyze the formation of a phosphodiester bond to repair the DNA damage [43,44]. Conversely, NC lesions are hardly repaired within the cell, and are usually produced by deoxyribonuclease II during programmed cell death pathways [45,46].

However, some pathways allowing the reparation of NC strand breaks exist. As an example, mammalian polynucleotide kinase adds a phosphate group at the 5′ position of non-canonical terminations while replacing the -PO_4_^−^ moiety at the 3′ position with a hydroxyl group, hence permitting the further action of DNA ligase [47,48]. In addition, some organisms have developed proper mechanisms for repairing non-canonical strand breaks. One example is the repair pathway involving RNA ligase RtcB in *Escherichia coli* or HD-Pnk in *Deinococcus radiodurans* [49,50].

The presence of G4s may induce strand break formation but also may oppose resistance to strand breaks. For example, if G4s cannot be unfolded by helicases, DNA replication is stopped and DNA breaks may occur at their location [51,52]. Conversely, they are also known to play an important role in radio-resistance and the response to DNA damage [25,53]. Recently, Kumari et al. [53] experimentally demonstrated the resistance of G4s to ionizing radiation and their presence in coding DNA sequence (CDS), although this resistance could also be ascribed to a shielding effect of the DNA backbone. Despite their interesting features, the impact of the occurrence of strand break lesions in G4s have been poorly documented from a structural and atomistic point of view.

Here, we investigate the structural effects of the presence of strand breaks in G4-forming DNA sequences containing both CA and NC lesions using state-of-the-art molecular modelling and simulation techniques. In particular, molecular dynamics (MD) simulations have been performed to check how the number and position of strand breaks affect the structure of a parallel G4 structure with a human telomeric sequence (h-telo). Our results show that G4 structures are extremely resistant to strand-breaks, an occurrence which may be correlated to a possible protective role exerted in conditions of high ionizing or oxidative stress.

## 2. Results

**Figure 2:**
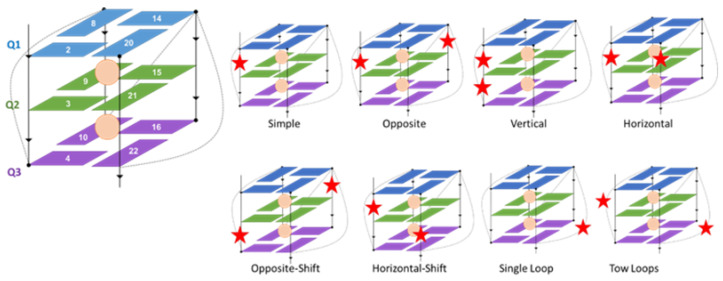
Position of tetrads in the studied h-telo G4 and the relative orientation of the strand break damage (displayed as red stars) in peripheral loops or the tetrad-connecting backbone. The orange dots represent cations.

A parallel h-telo G4 DNA was used as our model due to its presence in cells where it protects telomeres by acting as a telomerase inhibitor [54]. Although the h-telo sequence is characterized by high polymorphism, hybrid conformations are also possible [55], the parallel arrangement represents a suitable model for assessing stability in the presence of strand break lesions. Furthermore, we have previously shown that hybrid sequences show even higher stability to oxidative lesions than parallel arrangements [31]. In addition to the undamaged quadruplex used as a reference, 27 different structures harboring strand breaks have been taken into account. For both CA and NC forms, six single-breaks and six double-breaks have been introduced into the tetrads. Breaks, in CA forms, have also been introduced into the loops at three different positions, as shown in Table 1 and Figure 2. This choice allowed us to investigate the role of the position of the strand breaks on the structural stability of G4s. Note that the chosen strand break pattern follows a similar scheme used by us to study the impact of 8-oxoG on the structural stability of G4s [56]. We would also like to emphasize that in the present contribution, the word double-break refers to the presence of two cuts in a quadruplex sequence. Hence, it should not be confused with the usual double-strand nomenclature for B-DNA that refers to nearby breaks in two complementary strands. 

The presence of strand breaks inevitably induces some structural variations impacting the G4, which may be more or less marked, highly localized, or conversely, more global. Monitoring the evolution of the main structural parameters allowed us to account for the impact of the type of lesion and its position on the specific G4. Namely, we focused on the distance for the centers of mass of the tetrads, their twist angle, and the angles formed between guanines belonging to the same quartet. In addition, the RMSD of the guanines forming the tetrads highlights conservation of the quartet arrangement and global preservation of the G4 conformation. 

**Figure 3:**
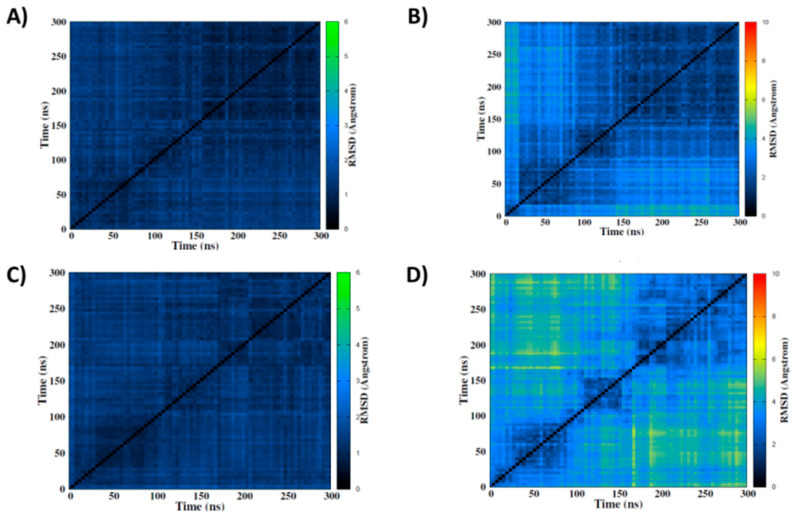
2D-RMSD maps of simulations of the native structure CA (top) and the 14-15-16 NC (bottom) including the tetrads only (**A**,**C**) or the whole DNA (**B**,**D**).

As shown in the Supplementary Materials (Figures S1–S56) and in Figure 3, RMSD clearly highlights conservation of the quartet arrangement of the G4, and hence the stability of this conformation. This is particularly visible in the 2D-RMSD maps plotted for each structure (Figure 3 and Figures S1–S56). Indeed, the structural deviation of the quartets remained relatively small throughout the simulation, rarely exceeding 3 Å. However, when considering the whole nucleic acid structure, we observed much larger structural variations, even for the native G4. This result is due to the peripheral loops, whose flexibility was clearly enhanced by the presence of strand breaks and constitutes further evidence for the coexistence of a rigid core with flexible loops in G4 arrangements. Interestingly, as shown in Figure 3 for specific strand break positions, this effect is similar and is produced analogously for both CA and NC structures. Remarkably, the stability of the G4 arrangement was also preserved in presence of multiple strand breaks, in contrast to what is observed for other lesions, such as abasic sites [56]. This is of great importance since ionizing radiation deposits its energy in a limited spatial area, usually leading to DNA cluster lesions [57,58].

The behavior of G4 is in striking contrast to canonical double-helical B-DNA structures. Indeed, in the latter case, a double-strand break induces high instability of the genome, which is mainly due to the dispersion of broken DNA fragments. Conversely, G4s appear to be much more resilient and strand breaks do not alter its arrangement. This feature can be correlated with the biological role of G4s and the protective role they can play in the presence of high oxidative stress. The global stability of G4s despite strand breaks also resonates with their resilience in the presence of oxidative damage, which we have recently determined [31]. However, it has to be pointed out that the guanine core may be much more sensitive to damage. Indeed, the introduction of an abasic site [56] may lead to G4 disruption or complex structural reorganization necessary to maintain its folding.

Our results agree with those reported by Kumari et al. [53], who experimentally demonstrated the resistance of G4s to strand breaks. Their in vitro experiments highlighted the formation of stable intra- and intermolecular G4s after exposure to ionizing radiation. In addition, their cell irradiation experiments suggested that G4-forming regions also exhibited high ionizing radiation resistance. Furthermore, their experimental results pointed to the fact that strand breaks occur mainly in G4-connecting loops. In fact, the introduction of a strand-break lesion into the loops caused them to open [53]. 

Such a situation undoubtedly leads to a modification of the global arrangement of G4 DNA. The phenomenon is particularly well highlighted by our 2D-RMSD maps (see Figures S1–S56). However, as the guanine core is not affected, the structural properties of the tetrads remained unchanged. Interestingly, from our simulations we may infer that strand breaks located in the connecting loops show an even higher structural resistance compared to those directly connecting the guanines that form the internal core. 

To provide a deeper analysis of the effects of strand break formation on G4 structures, we also considered more local deformations. As a matter of fact, Hoogsteen base-pairing of guanines and the interaction with alkali metal ions are crucial for the formation of G4s [59,60], whereas the involvement of the backbone should be considered minor for dictating their formation. 

The time series for the angles formed between the guanines in the tetrads still show a global stability (see Figure S57 and Table S1). More precisely, the values were all globally centered around the values measured in the native undamaged structure, i.e., ca. 90° for adjacent and ca. 180° for opposite guanines, with fluctuations being indicative of only slightly minor changes in their arrangement. If we focus on the respective distances between the center-of-mass of the tetrads, we observe only slight fluctuations compared to the native structure, in agreement with the global stability revealed by the RMSD analysis. 

**Figure 4:**
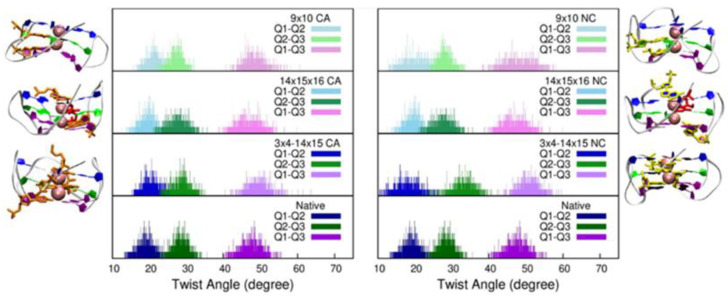
Distribution of the twist angle for representative DNA damaged structures (upper panels) compared to those of the native structure (bottom panels).

Instead, larger fluctuations were be observed for the twist angles (see Table S1 and Figure 4). Indeed, the presence of strand breaks induced a slight enlargement of their distribution, which may be related to increased flexibility. This is particularly evident for the twist angle between the two terminal tetrads, whereas variations in the twists involving the central tetrad were less important. In addition to enlargement of the distribution, we also noticed some deviations in the maximum value that may lead to a deviation between 5 and 10° from the undamaged structure, pointing to a slightly, albeit non-negligible, structural reorganization. This deviation was maximal when the lesion was in the backbone directly connecting two tetrads, and less pronounced when the loops were involved. 

Finally, and despite the global stability observed and discussed, we should point out that a significant exception was observed for one trajectory. This involved one replica for CA damage at the 14–15 position (Figure S10). In this case, we observed rearrangement of the first peripheral quartet that was accompanied by the leakage of a K^+^ cation, leading to the expulsion of the guanine G8 from a loop region and complete destabilization of the peripheral tetrad. In the past, we have observed that loss of the cation is an important phenomenon in the destabilization of the G4 structure [31,56]. Although this case highlights that metal cations are crucial role for the stability of G4 arrangements, it an remains isolated instance, since it was only observed once in all of our simulations and can therefore be considered a rare event.

## 3. Discussion and Conclusions

Strand breaks are important DNA lesions typically produced by exposure to ionizing radiation. Double-breaks in a DNA strand are difficult to repair and are associated with very high cytotoxicity. In this contribution, we investigated the impact of strand breaks on the stability and persistence of intra-strand G4 architecture, considering the important regulatory role played by G4-DNA structures at the cellular level. Although double-helical B-DNA strand breaks are usually correlated with strong structural destabilization and consequent genome dispersion, our results consistently showed that G4s experienced only negligible structural deformation in 26 out of 27 cases and maintained their global folding and shape. The introduction of a strand break is typically accompanied by a slight increase in flexibility of the connecting loops, and a slight change of the twist angle, especially when the break is directly located between guanines belonging to different tetrads. Only one strand break, i.e., between the 14th and 15th nucleotides, led to unfolding of the G4 in the second replica. This was due to the deformation of one tetrad and subsequent expulsion of the stabilizing K^+^ cation, which can be considered a rare event. Our results, which agree with those reported by Kumari et al. [53], confirm the high stability of G4s and their inherent resistance to strand-break damage. From a molecular point of view, this can be attributed to the combined effect of intra-tetrad Hoogsteen H-bonds (conferring rigidity to the tetrad core) and inter-tetrad π-stacking interactions (contributing to the maintenance of global G4 folding). From a cellular point of view, this occurrence can be related to the protective and regulatory role played by G4s in regulating gene expression and cellular senescence. As already noted for oxidative lesions, preserving G4 arrangement during conditions of high stress has the effect of limiting the expression of oncogenes, inhibiting telomerase, and avoiding the emergence of an immortal phenotype, i.e., phenomena that can lead to carcinogenesis.

It is important to point out that our MD simulations consistently used a pre-folded G4 on top of which strand breaks were created. Hence, our results are unequivocal concerning the stability of the quadruplexes. However, we could not infer whether the presence of strand breaks could inhibit the folding itself, and hence, the presence of actual G4 in cells. The propensity for G4 folding of damaged DNA, which might also strongly depend on the length of the fragments, and hence, radiation intensity, will require enhanced sampling procedures that will be addressed in forthcoming contributions.

## 4. Materials and Methods

### 4.1. Force Field for Non-Standard Nucleotides 

Prior to modelling the formation of strand breaks, specific force field parameters for the two sides of the cleaved backbone needed to be generated. To this aim, we chose the AMBER ff99bsc1 force field [61,62] and specific modifications to the guanine and adenine force field were performed to obtain CA and NC ends (see Supplementary Materials). The geometry of each of the new residues was optimized at the B3LYP/6-311+G(d,p) level of theory, with Gaussian 09 [63]. Restrained electrostatic potential (RESP) charges were obtained at HF/6-31G * and converted into amber format with the antechamber utilities. 

### 4.2. Molecular Dynamics Simulations

All simulations were generated using the AMBER16 suite of programs [64]. The initial G4 structure of h-telo was obtained from the pdb database (PDB:1KF1) [65], and the strand breaks were manually created at specific sequence positions (see Table 1 and ESI). Then, the initial systems were solvated in an octahedral TIP3P water box [66] with a 12 Å buffer, and electroneutrality was provided by the addition of K^+^ ions. Note that the central K^+^ ions present in the crystal structure have been kept. Hydrogen mass repartitioning [67] was applied to allow the integration of the Newton equations of motion using a 4 fs time step in combination with the RATTLE and SHAKE algorithms [68]. All MD simulations were performed with NAMD software [69,70] until a simulation time of 300 ns was reached in the NPT ensemble maintained by a Langevin thermostat and barostat [71]. Each of simulation was preceded by 1000 minimization steps and 36 ns of equilibration. All simulations were performed on two independent replicas to increase global sampling. VMD [72] was used to visualize and analyze the MD trajectories. G-quadruplex structural parameters were calculated using the script developed by Tsvetkov et al. [73].

## Figures and Tables

**Table 1:** Schematic representation of the positions of the considered strand breaks. as indicated by the symbol ★. CA and NC strand breaks occur on the two O-P(O2)-O sides of the same phosphate group.

Strand Break Type	Strand Break Position	Sequence
Native		A GGG TTA GGG TTA GGG TTA GGG
Single	2–3	A G★GG TTA GGG TTA GGG TTA GGG
	3–4	A GG★G TTA GGG TTA GGG TTA GGG
	9–10	A GGG TTA GG★G TTA GGG TTA GGG
	14–15	A GGG TTA GGG TTA G★GG TTA GGG
	15–16	A GGG TTA GGG TTA GG★G TTA GGG
	21–22	A GGG TTA GGG TTA GGG TTA GG★G
Double	2–3–4	A G★G★G TTA GGG TTA GGG TTA GGG
	2–3/14–15	A G★GG TTA GGG TTA G★GG TTA GGG
	3–4/9–10	A GG★G TTA GG★G TTA GGG TTA GGG
	3–4/14–15	A GG★G TTA GGG TTA G★GG TTA GGG
	3–4/15–16	A GG★G TTA GGG TTA GG★G TTA GGG
	14–15–16	A GGG TTA GGG TTA G★G★G TTA GGG

The symbol ★ indicates the position of the CA and NC strand breaks occurring on the two O-P(O2)-O sides of the same phosphate group.

## Data Availability

Trajectories of the MD simulations are available upon request.

## Supplementary Materials

The following supporting information can be downloaded at: https://www.mdpi.com/article/10.3390/molecules27103256/s1, full methodological details and structural analysis of the different G4 strands.
